# An Innovative Mei-Gin Formula Exerts Anti-Adipogenic and Anti-Obesity Effects in 3T3-L1 Adipocyte and High-Fat Diet-Induced Obese Rats

**DOI:** 10.3390/foods12050945

**Published:** 2023-02-23

**Authors:** Hsin-Lin Cheng, Wei-Tang Chang, Jiun-Ling Lin, Ming-Ching Cheng, Shih-Chien Huang, Shiuan-Chih Chen, Yue-Ching Wong, Chin-Lin Hsu

**Affiliations:** 1 Department of Nutrition, Chung Shan Medical University, Taichung 40201, Taiwan; 2 Department of Nutrition and Health Sciences, Chinese Culture University, Taipei 11114, Taiwan; 3 Department of Nutrition, Chung Shan Medical University Hospital, Taichung 40201, Taiwan; 4 Department of Health Food, Chung Chou University of Science and Technology, Changhua 51591, Taiwan; 5 Department of Health Industry Technology Management, Chung Shan Medical University, Taichung 40201, Taiwan; 6 Institute of Medicine and School of Medicine, Chung Shan Medical University, Taichung 40201, Taiwan; 7 Department of Family and Community Medicine, Chung Shan Medical University Hospital, Taichung 40201, Taiwan

**Keywords:** Mei-Gin formula, anti-obesity, 3T3-L1, high-fat diet, Wistar rats

## Abstract

Background: To investigate the potential anti-obesity properties of an innovative functional formula (called the Mei-Gin formula: MGF) consisting of bainiku-ekisu, *Prunus mume* (70% ethanol extract), black garlic (water extract), and *Mesona procumbens* Hemsl. (40% ethanol extract) for reducing lipid accumulation in 3T3-L1 adipocytes in vitro and obese rats in vivo. Material and Methods: The prevention and regression of high-fat diet (HFD)-induced obesity by the intervention of Japan Mei-Gin, MGF-3 and -7, and positive health supplement powder were investigated in male Wistar rats. The anti-obesity effects of MGF-3 and -7 in rats with HFD-induced obesity were examined by analyzing the role of visceral and subcutaneous adipose tissue in the development of obesity. Results: The results indicated that MGF-1-7 significantly suppressed lipid accumulation and cell differentiation through the down-regulation of GPDH activity, as a key regulator in the synthesis of triglycerides. Additionally, MGF-3 and MGF-7 exhibited a greater inhibitory effect on adipogenesis in 3T3-L1 adipocytes. The high-fat diet increased body weight, liver weight, and total body fat (visceral and subcutaneous fat) in obese rats, while these alterations were effectively improved by the administration of MGF-3 and -7, especially MGF-7. Conclusion: This study highlights the role of the Mei-Gin formula, particularly MGF-7, in anti-obesity action, which has the potential to be used as a therapeutic agent for the prevention or treatment of obesity.

## 1. Introduction

Obesity is characterized by a defective body fat storage capacity caused by a chronic imbalance of energy due to excess dietary consumption and insufficient physical activity [1]. The prevalence of obesity is still rising globally and has become a pervasive public health threat. Obesity precedes type 2 diabetes mellitus, dyslipidemia, fatty liver injury, hypertension, and cancer, and is greatly associated with a higher premature disability and mortality rate [2]. Excess calorie intake is accompanied by less energy expenditure, leading to adipogenesis in both the liver and the adipose tissue, and subsequently promoting the development of metabolic disorders [3,4].

White adipose tissue (WAT) represents a key reservoir for energy storage such as triglycerides (TG) in adipose tissue and expands via increasing individual size (hypertrophy) or number (hyperplasia) of differentiated mature adipocytes to allow adequate tissue expansion in response to high-fat dietary consumption or overnutrition [5,6,7]. The adipocyte differentiation process begins from adipocyte progenitors’ mitogenic expansion in the determination phase, and later gains the characteristics of mature adipocytes in the terminal differentiation phase. Anatomically, subcutaneous adipose tissue (SAT) and visceral adipose tissue (VAT) are considered the two main types of WAT; the enlargement of the SAT and VAT enlargement are considered to mediate obesity development and its related metabolic complications [8,9,10,11]. In particular, rodents were fed a high-fat diet with the consequence of a significant imbalance in energy storage and expenditure capacity between WAT and brown adipose tissue (BAT) depots [12,13]. Evidence from animal and human studies indicates that the inability of SAT and VAT to expand when faced with dietary phytochemicals is pharmacologically beneficial to the metabolic health status of obesity [14,15,16]. Lipid metabolism is often considered a complex mechanism with the involvement of regulatory elements such as glycerol-3-phosphate dehydrogenase (GPDH), which mediate the rate-determining reaction in the synthesis of triglycerides and serve as a marker of adipogenesis [17]. Numerous studies indicated that inhibition of GPDH expression or activity can suppress lipid accumulation in 3T3-L1 adipocytes and may act as an anti-obesity target in adipocytes [17,18,19,20].

Bainiku-ekisu is the fruit juice concentrate of *Prunus mume* and has been proposed to have pharmacological properties that might benefit the treatment of dyspepsia and diarrhea. An in vitro study has shown that bainiku-ekisu exhibited immediate bacteriostatic activity on serious strains of *H. pylori* at a concentration of 0.3% in 15 min [21]. Furthermore, an in vivo pilot study reported that *H. pylori*-positive patients received 1% bainiku-ekisu solutions for 2 weeks, resulting in a slightly decreased in the urea breath test (UBT) values [22]. Yang et al. demonstrated that bainiku-ekisu has a higher total phenolic content (1.9-fold) and flavonoid content (1.4-fold) than fresh Japanese apricot juice, which may have direct effects on improving metabolic disorders, and, therefore, improve metabolic diseases such as type 2 diabetes and hyperlipidemia [23]. Black garlic, a fermented product of fresh garlic, is generated by the application of controlled high temperature under high humidity over 10 days [24,25]. A previous study suggested that fermented black garlic exhibited bioprotective properties against vascular disease through the downregulation of the MAPK pathway in a model of zebrafish vascular lesions [26]. Extracts from black garlic decreased body weight (7.69%) and lumbar subcutaneous fat mass (16.88%) in a high-fat diet-fed rodents model [27]. Treatment with black garlic extract was founded to increase cellular oxygen uptake and alter UCP-1-based thermogenesis in human adipose-derived stem cells [28]. *Mesona procumbens* Hemsl., also called Hsian-tsao, is consumed as folk medicine and is considered to have therapeutic potential for the treatment of liver disease, heat shock, and metabolic disorders. The anti-adipogenic activity of *Mesona procumbens* in 3T3-L1 cells was also reported to decrease lipid droplet accumulation by transcriptionally inhibiting peroxisome proliferator-activated receptor γ (PPARγ) and transcription factors CCAAT/enhancer-binding protein (C/EBP) β expressions [29]. We have previously shown that in vivo and in vitro, the *Mesona procumbens* Hemsl. extract could decrease xanthine oxidase activity and prevent the overproduction of serum uric acid, which is suggested to be a novel hypouricemic agent [30]. In addition, extracts rich in phenolic compounds from Hsian-tsao have been demonstrated to exhibit antioxidant properties and served as free radical scavengers [31].

Based on the accumulated experimental evidence, we have developed an innovative bainiku-ekisu-based functional combination of ethanol extracts from *Prunus mume*, *Mesona procumbens* Hemsl., and water extract from black garlic, called the Mei-Gin formula (MGF). However, due to the bioavailability of individual substances in the complex mixture and differences in the pharmacokinetics of the active substance, a deeper understanding of the beneficial effect of the Mei-Gin formula in the prevention or treatment of metabolic parameters in obesity is now an urgent need. In the present study, our aim is to investigate the efficacy of the Mei-Gin formula in pre-adipose cell differentiation and a high-fat diet-fed rat model.

## 2. Materials and Methods

### 2.1. Composition Analysis of the Mei-Gin Formula (MGF)

MGF-1-7 capsules containing mixed extract of functional powder, consisting of bainiku-ekisu (decompress concentration process), *Prunus mume* (70% ethanol extract), black garlic (water extract), and Hsian-tsao (40% ethanol extract) were provided by Dr. Ming-Ching Cheng, Department of Healthy Food, Chung Chou University of Science and Technology. In brief, analysis of the phenolic content of the Mei-Gin formulas was carried out using a high-performance liquid chromatography system (L-2130 pump and L-2400 UV detector, Hitachi, Tokyo, Japan), and permeation data were recorded on a computer with the LC solution 1.25 sp1 software. Elution was carried out with two buffers: buffer A (0.1% formic acid in water) and buffer B (0.1% formic acid in acetonitrile); the flow rate was set at 1 mL/min. The absorption spectra of the samples were detected at a UV wavelength of 280 nm, and all phenolic acid identification was carried out by comparing their retention time with known reference standards [32].

### 2.2. Cell Culture and Treatment

3T3-L1 preadipocytes (BCRC No. 60159) obtained from the Bioresource Collection and Research Center (BCRC, Food Industry Research and Development Institute, Hsinchu, Taiwan) were cultured in high glucose Dulbecco’s modified Eagle medium (high glucose DMEM) supplemented with 9% newborn calf serum (NBCS), NaHCO_3_ (1.5 g/L) and 1% penicillin-streptomycin (PSN) in an incubator with an atmosphere of 5% CO_2_. For the differentiation of adipocytes, 3T3-L1 cells were cultured in the differentiation medium containing 0.5 mM 3-isobutyl-1-methylxanthine (IBMX), 1 μM dexamethasone (DEX), 1 μM insulin, 1.5 g/L NaHCO_3_, and 1% PSN. After 4 days of incubation, the medium was changed to high glucose DMEM containing 8% fetal bovine serum (FBS), 1 μM insulin, 1.5 g/L NaHCO_3_, and 1% PSN for 2 days. The mature 3T3-L1 adipocytes were treated with appropriate doses of MGF-1-7 solution (0, 10, 25, 50, 100, and 250 μg/mL) and then incubated for 48 h.

### 2.3. Animals and Diet

**Figure 1:**
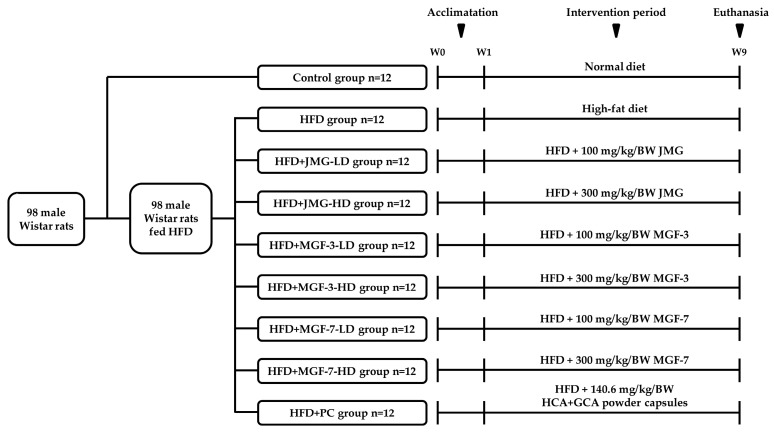
Flow diagram of the study on the anti-obesity effects of the Mei-Gin formula in rats with HFD-induced obesity.

Six-week-old male Wistar rats were purchased from BioLASCO Taiwan Co., Ltd. (Taipei, Taiwan) and supplied with a pelletized commercial laboratory diet (Purina Lab Chow) and water ad libitum. The rats were maintained under an air-conditioned environment (23 ± 2 ℃ with 60% relative humidity) with a 12 h light/dark cycle at the Experimental Animal Center of Chung Shan Medical University. All the experimental manipulations involving animals were strictly implemented according to ethical guidelines for animal experiments and were approved by the Laboratory Animals Center of Chung Shan Medical University (IACUC No. 1664). After 1 week of acclimatization, the rats were randomly divided into nine groups of 12 rats (Figure 1). Rats in the control group were fed an AIN-93G control diet (7% fat); rats in the experimental groups were fed an AIN-93G-based high-fat diet containing 32% lipids (7% soybean oil and 25% lard). For the positive control treatment, a health supplement containing hydroxycitric acid (HCA) and chlorogenic acid (CGA) was obtained from Taiyen Biotech Co., Ltd. (Tainan, Taiwan), which has been proven to have antibody fat accumulation effects by the Taiwan Food and Drug Administration (TFDA, License NO. A00274). Furthermore, the experimental groups were distributed into eight subgroups: (a) continued received HFD throughout the period (HFD), (b) HFD supplemented with 100 mg/kg body weight Japan Mei-Gin (low-dose Japan Mei-Gin, HFD + JMG-LD), (c) HFD supplemented with 300 mg/kg body weight Japan Mei-Gin (high-dose Japan Mei-Gin, HFD + JMG-HD), (d) HFD supplemented with 100 mg/kg body weight Mei-Gin formula-3 (low-dose Mei-Gin formula-3, HFD + MGF-3-LD), (e) HFD supplemented with 300 mg/kg body weight Mei-Gin formula-3 (high-dose Mei-Gin formula-3, HFD + MGF-3-HD), (f) HFD supplemented with 100 mg/kg body weight Mei-Gin formula-7 (low-dose Mei-Gin formula-7, HFD + MGF-7-LD), (g) HFD supplemented with 300 mg/kg body weight Mei-Gin formula-7 (high-dose Mei-Gin formula-7, HFD + MGF-7-HD), and (h) HFD supplemented with 140.6 mg/kg body weight HCA + GCA powder capsules (HFD + PC). During the experiment period, the body weight, daily feed, and water were measured and used to calculate the feeding efficiency. After 8 weeks, animals were fasted overnight, euthanized by carbon dioxide, and whole blood was collected from the abdominal aorta. The heart, liver, spleen, lung, kidney, visceral adipose tissue (perirenal fat, epididymal fat, and mesenteric fat), and subcutaneous adipose tissue (retroperitoneal fat and inguinal fat) were dissected, rinsed, weighed, and stored at −80 °C.

### 2.4. Oil Red O Lipid Staining

For measuring intracellular lipid accumulation, oil red O staining was performed to determine the effect of MGF-1-7 on lipid synthesis. Briefly, mature 3T3-L1 adipocytes were harvested and then washed by PBS and fixed in 10% neutral buffered formalin for 20 min at room temperature. Subsequently, cells were placed in 100% propylene glycol for 3 min and then stained with a mixture of oil red O working solution (oil red O solution/water 3:2, *v/v*) for 60 min. The lipid droplets were visualized and photographed by microscopy (Motic AE30/31), and the oil red O was solubilized in isopropanol and measured spectrophotometrically at 510 nm.

### 2.5. Determination of Glycerol-3-Phosphate Dehydrogenase (GPDH) Activity

Using a GPDH activity colorimetric assay kit (Cat No. K640-100, BioVision, Milpitas, CA, USA), mature 3T3-L1 adipocytes were harvested 48 h after MGF-1-7. Based on the manufacturer’s instructions, protein concentration was determined spectrophotometrically according to the reduced nicotinamide adenine dinucleotide (NADH) standard. GPDH activity (%) was pressed as a percentage change against control (100%).

### 2.6. Statistical Analysis

For all experiments, the quantitative data are expressed as mean ± SEM. The analysis of variance was followed by one-way ANOVA with Duncan’s multiple range test by using the SPSS software program, version22.0 (APSS Inc., Chicago, IL, USA). A statistically significant difference was established only if the *p*-value < 0.05.

## 3. Results

### 3.1. Determination of Phenolic Contents of the Mei-Gin Formulas

The content of the main phenolic compounds in MGF-1-7 was identified using HPLC analysis and as follows: MGF-1 (chlorogenic acid: 0.46, caffeic acid: 0.19, and *p*-coumaric acid: 0.09 mg/g), MGF-2 (chlorogenic acid: 0.26, caffeic acid: 0.24, and *p*-coumaric acid: 0.07 mg/g), MGF-3 (chlorogenic acid: 0.34, caffeic acid: 0.18, and *p*-coumaric acid: 0.08 mg/g), MGF-4 (chlorogenic acid: 0.32, caffeic acid: 0.26, and *p*-coumaric acid: 0.08 mg/g), MGF-5 (chlorogenic acid: 0.54, caffeic acid: 0.11, and *p*-coumaric acid: 0.08 mg/g), MGF-6 (chlorogenic acid: 0.70, caffeic acid: 0.19, and *p*-coumaric acid: 0.07 mg/g), and MGF-7 (chlorogenic acid: 0.72, caffeic acid: 0.11, and *p*-coumaric acid: 0.08 mg/g). The data indicated that MGF-7 had higher chlorogenic acid, MGF-4 had higher contents of caffeic acid content, and the contents of *p*-coumaric acid content was similar in each group.

### 3.2. Effects of Mei-Gin Formulas on Lipid Accumulation in 3T3-L1 Adipocytes

**Figure 2:**
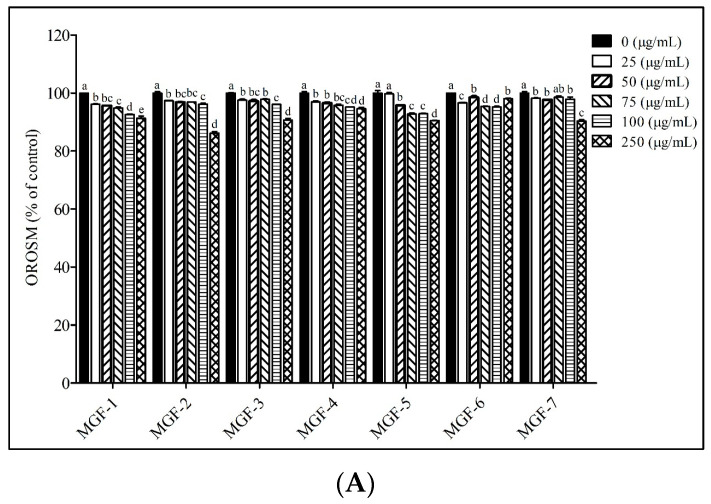

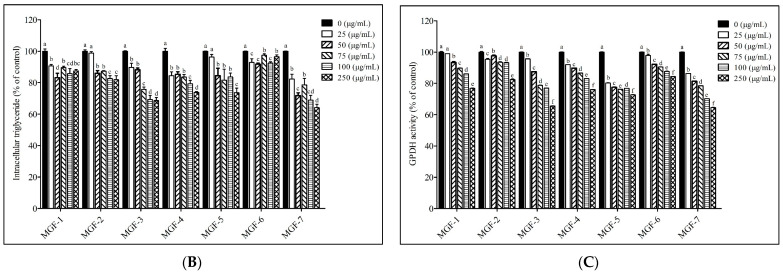
Effects of MGF-1-7 on the adipogenesis in 3T3-L1 adipocytes. (**A**) Quantification of the lipid droplets in 3T3-L1 adipocytes stained with oil-red O-stained material (OROSM). 3T3-L1 adipocytes were treated with MGF-1-7 (0–250 μg/mL) for 48 h at 37 °C in a 5% CO_2_ incubator. (**B**) Quantification of intracellular triglyceride levels in 3T3-L1 adipocytes. (**C**) Relative activity of GPDH in 3T3-L1 adipocytes. All values are presented as mean ± SEM. The percentage (%) of variables was estimated by comparing it with the control group. The values with different letters indicated a significant change between groups as determined by one-way ANOVA with post hoc Duncan’s test. Differences were considered statistically significant when *p* < 0.05.

To determine the effects of the Mei-Gin formula on intracellular lipid accumulation in adipocyte cells, the effects of serially diluted MGF-1-7 on 3T3-L1 adipocytes were visualized using oil red O staining. MGF-1-7 effectively reduced lipid accumulation in mature 3T3-L1 adipocytes. To validate the observation of reduced lipid accumulation, a TG quantification assay was performed to confirm the changes. As shown in Figure 2A,B, similar to the reduction of lipid accumulation, MGF-1-7 significantly decreased the cellular level of TG in 3T3-L1 adipocytes. The 250 μg/mL MGF-3 and -7 treatments were observed to show a significant 31.4 and 35.9% reduction in TG content compared to untreated control cells, respectively (Figure 2B), indicating that in the presence of high-dose MGF-3 and -7, the intracellular level of TG levels was dramatically decreased. Additionally, a further detailed analysis of the effects of MGF-1-7 on GPDH activity is shown in Figure 2C. The alteration of intracellular GPDH activity was observed in 3T3-L1 adipocytes treated with MGF-1-7. In particular, GPDH activity was decreased in the same manner as previously observed in the intracellular TG level in cells treated with MGF-3 and -7. The results suggested that MGF-3 and -7 appeared to have the most potency in reducing 3T3-L1 adipocytes. Therefore, we used MGF-3 and -7 in subsequent experiments involving animal models.

### 3.3. Effect of the Mei-Gin Formula on Body Weight, Feed Intake, Energy Intake, Feed Efficiency, and Mass of Selected Organs in HFD-Induced Obesity

**Figure 3:**
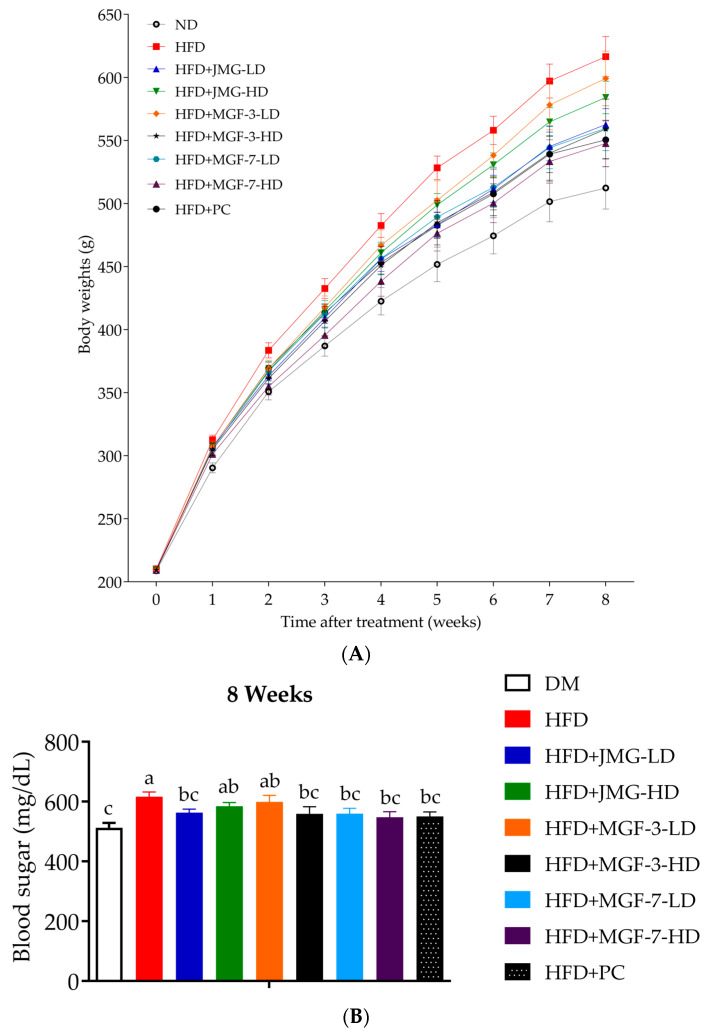
Effects of MGF-3 and -7 on body weight in high-fat diet-induced obese rats. (**A**) Body weight change during the experimental period; (**B**) body weight at week 8. All values are presented as mean ± SEM. The values with different letters indicated a significant change between groups as determined by one-way ANOVA with post hoc Duncan’s test. Differences were considered statistically significant when *p* < 0.05. ND: normal diet; HFD: high-fat diet; HFD + JMG-LD: HFD treated with low-dose Japan Mei-Gin; HFD + JMG-HD: HFD treated with high-dose Japan Mei-Gin; HFD + MGF-3-LD: HFD treated with low-dose Mei-Gin formula-3; HFD + MGF-3-HD: HFD treated with high-dose Mei-Gin formula-3; HFD + MGF-7-LD: HFD treated with low-dose Mei-Gin formula-7; HFD + MGF-7-HD: HFD treated with high-dose Mei-Gin formula-7; HFD + PC: HFD treated with positive control. Weight change (g) = final body weight (g) − initial body weight (g).

As shown in Figure 3 and Table 1, the body weight during the experimental period progressively decreased among the JMG, MGF, and PC intervention groups, as compared to the HFD group. In detail, consumption of low-dose Japanese MG, high (300 mg/kg) MGF-3, and both low and high (100 mg/kg and 300 mg/kg) MGF-7 significantly decreased body weight change and weight gain compared to that of the HFD group (*p* < 0.05). The feed intake and energy intake of the JMG-fed rats were significantly lower than those of the HFD-fed rats. Although the feed efficiency of rats that consumed MGF-3 (300 mg/kg), MGF-7 (300 mg/kg), and HCA + GCA powder capsules (140.6 mg/kg) was significantly lower than that of the HFD group (Table 2), no significant differences were observed in the weights of the heart, spleen, lungs, and kidneys among each group. A significant reversal in increased liver weight was observed in rats that ate low (100 mg/kg) JMG, high (300 mg/kg) MGF-3, low and high (100 mg/kg and 300 mg/kg) MGF-7, and powder capsules (140.6 mg/kg) (Table 3).

### 3.4. Effect of the Mei-Gin Formula on Body Fat Mass and Adipose Tissue in Rates with HFD-Induced Obesity Rates

As shown in Table 4 and Table 5, the rats fed a high-fat diet exhibited persistent higher total body fat mass compared to the counterpart ND group, and this was significantly attenuated by consuming low (100 mg/kg) JMG, high (300 mg/kg) MGF-3, both low and high (100 mg/kg and 300 mg/kg) MGF-7, and HCA + GCA powder capsules (140.6 mg/kg). Similarly, a trend of reduction in subcutaneous adipose tissue was also observed among those five groups as the result of increased retroperitoneal and inguinal adipose tissue. Although the effect of low-dose JMG on visceral adipose tissue was not statistically different, it still showed potential to lower the mass of adipose tissue at week 8. Among visceral (perirenal, epididymal, and mesenteric) adipose tissue, a significant reduction was found in perirenal and mesenteric adipose tissue after administration of low-dose JMG, high-dose MGF-3, both high- and low-dose MGF-7, and powder capsules; while the weight of epididymal adipose tissue did not show a significant difference between each group after dietary intervention.

## 4. Discussion

Obesity is characterized by defective excess body fat content, including the determination of size and body fat distribution. Recently, emerging evidence has focused on dietary phenolic compounds that provide a therapeutic strategy for people with obesity, as naturally occurring plant products reduced the potential for side effects [33,34,35,36]. Concerning the risk of adverse medication reactions, various options for obesity management and treatments have been constantly conducted in various biological properties of natural phenolic compounds that exhibited a preventive or therapeutic potential to improve lipid and glucose dysregulation [37,38,39]. Existing anti-obesity medications including orlistat and sibutramine, with very modest efficacy, can cause clinically adverse drug reactions. Therefore, there is a strong need to exploit and discover naturally occurring foods and substances as safe and acceptable alternatives to designer drugs. Based on Chinese herbology, we developed a novel Mei-Gin formula to exert a powerful synergistic effect to target the development of obesity [40,41]. In this study, our aim was to determine the anti-obesity effect of the Mei-Gin formula on the 3T3-L1 cells in vitro and in vivo HFD-induced rat model by monitoring the regulation of cell lipid accumulation and adipose tissue. In vitro cell models, particularly 3T3-L1 preadipocyte differentiation and adipogenesis of 3T3-L1 preadipocytes, with the main characteristic of intracellular triglyceride accumulation, are associated with the development of obesity [42,43]. In this regard, we demonstrated that the inhibitory effects of the Mei-Gin formula on 3T3-L1 adipocyte differentiation were due to the downregulation of GPDH activity and this subsequently led to a reduction in cellular triglyceride production. As shown in Figure 2, including the serial tested Mei-Gin formulas (MGF-1-7), all are capable of altering the differentiation capacity of the 3T3-L1 preadipocyte into the 3T3-L1 adipocyte. On the basis of the HPLC analysis, we determined the phenolic compounds in the Mei-Gin formula which were most abundant in p-coumaric acid, caffeic acid, and chlorogenic acid. Among food phenolic compounds, hydroxycinnamic acid represents a major class of phenolic acid available in fruits, seeds, and vegetables [44,45]. A diet supplement of hydroxycinnamic acid can easily reach levels of 0.5–1 g or even higher in humans. Previously, evidence indicated that hydroxycinnamic acid, including *p*-coumaric acid caffeic acid, ferulic acid, and chlorogenic acid, can serve as the primary antioxidant [46], have powerful anti-inflammatory [47] and anti-cancer activity [48,49], and are involved in improving insulin resistance [50]. The study reveals that caffeic acid phenethyl ester effectively prevents body weight gain and the gain of epididymal adipose tissue [51]. The result of oil red O staining indicated that caffeic acid phenethyl ester significantly reduced adipogenesis in 3T3-L1 preadipocytes, which is in accordance with our in vivo results. Dietary consumption of chlorogenic acid markedly altered the plasma lipid profile and attenuated the fatty liver by increasing hepatic PPAR-α expression in hypercholesterolemic rats [52]. Furthermore, *p*-coumaric acid-induced activation of the AMPK pathway subsequently leads to the inhibition of adipogenesis in 3T3-L1 adipocytes [53]. To understand the synergistic effects for each of the natural plants in our new formula, different ratios of each individual plant were used to form MGF-1-7 in the context. Interestingly, quantitative results from intracellular triglycerides in 3T3-L1 adipocytes indicated that MGF-3, -4, and -7 exhibited predominant inhibitory effects on lipid accumulation, while the inhibitory capacity among Mei-Gin formulas on GPDH activity was found mostly in MGF-3, -5, and -7. Taken together, MGF-3 and -7 appeared to be the most potent in regulating the development of obesity. Therefore, we examined the effectiveness of MGF-3 and -7 to ameliorate weight gain and further identified the contribution of adipose tissues to obesity in the HFD-induced rat model.

Previously, rats fed a high-fat/high-energy diet have been shown to have significantly increased body weight compared to normal diet-fed rats, and are widely used in diet-induced obesity studies [54,55]. In the rat model induced by a high-fat diet, excessive lipid accumulation in subcutaneous and visceral adipose tissue is a key feature of obesity. In the present study, we observed that the administration of the Mei-Gin formula in obese rats for 8 weeks significantly reduced final body weight and weight gain. Furthermore, both low- and high-dose MGF-7 effectively suppressed body weight compared to MGF-3. Importantly, diet-induced obesity is associated with lipid burden in adipose tissue, as well as in non-adipose tissue. The increased fat deposition was observed in the liver and eventually leads to weight gain in the obese animal model [36,56,57,58]. Our data reveal a similar trend of reduction of liver weight in HFD-induced rats after administration of the Mei-Gin formula. Interestingly, a similar result was shown in 3T3-L1 adipocytes in vitro and obese mice in vivo as a supplement of plant resin [59]. Recently, the complete analysis of *Prunus mume* extracts was reported to identify the phytochemical composition, including chlorogenic acid, lupeol, mumefural, and ursolic acid, which are proposed to have anti-cancer properties [60,61,62,63]. A pilot study conducted on 18 *H. pylori*-positive participants in the stomach proposed an anti-bacterial activity of bainiku-ekisu therapy [22]. Bainiku-ekisu is a *Prunus mume* juice concentrate and was previously reported to exhibit strong anti-bacterial activity in vitro [23]. Due to the concentration process, the phytochemical contents such as phenolic acid and flavonoids were higher in bainiku-ekisu than those in *Prunus mume* juice [64,65]. Herein, we are the first to verify the effects of the Mei-Gin-based plant mixture in regulating body weight gain of obese rats. High-performance liquid chromatography analysis confirmed the phenolic acid and flavonoid constituents in the thermal process and was found to increase markedly in black garlic as compared to that of fresh garlic [66,67,68]. The HPLC isolation and identification of black garlic showed variable quantities of phenolic acid, including garlic acid, vanillic acid, chlorogenic acid, caffeic acid, *p*-coumaric acid, and ferulic acid. Hung et al. have investigated the antioxidant activity and active components of Hsian-tsao [69]. Several phenolic acids were identified from the water extract of Hsian-tsao, including apigenin, caffeic acid, vanillic acid, and kaempferol [70]. The authors also determined that the amounts of caffeic acid and kaempferol were the highest among those phenolic acids and are considered the main functional components, and may contribute to the antioxidant properties of Hsian-tsao. The diet supplement of phenolic compounds from natural plants that have served as potent anti-obesity agents has been well documented. However, the effects of natural plants on obesity are not yet defined. The composition of the innovative plant formula was based on the hypothesis that combining the candidates of the plants with particular reference mentioned above can modulate the development of obesity in vivo and in vitro. Our data demonstrated that low-dose JMG, high-dose Mei-Gin formula, both high-and low-dose Mei-Gin formula, and HCA + GCA powder capsules effectively reduced total body fat in HFD-induced rats, which is consistent with the initial observation in body weight gain and liver weight. In addition, we identified the contribution of adipose tissue in obese rats. Along the same lines, it leads to a significant reduction in visceral (perirenal and mesenteric adipose tissue) and subcutaneous (retroperitoneal and inguinal adipose tissue) adipose compartments in HFD-induced rats compared to HFD controls. These data demonstrated that the administration of the Mei-Gin formula caused weight loss, which affected both visceral and subcutaneous adipose tissue. However, no differences were established regarding low-dose JMG and HFD controls.

JMG is a condensed extract obtained using traditional thermal condensation methods. Accumulated studies have shown that JMG inhibits the proliferation of hepatocellular carcinoma cells [71] and improves hyperglycemia [72], which can serve as a dietary intervention as adjuvant therapy. However, phytochemical compounds such as phenolic acid have been reported to be destroyed after heat treatment at high temperatures for a long period of time [64,65]. In the present study, high-dose MGF-3 and high- and low-dose MGF-7 administration in HFD-induced rats results in a more efficient reduction in body weight gain, liver weight, and total body fat compared to JMG groups, which may be attributed to our decompression-processed Mei-Gin, which kept more bioactive components. Among each group, the optimal level of each MG formula group has been determined in the corresponding HFD-induced rat model. The results suggested that high-dose MGF-7 exerts the most potent anti-obesity activity.

## 5. Conclusions

In conclusion, this is the first study to verify the effects of a Mei-Gin-based plant formula on obesity both in vivo and in vitro. MGF-1-7 reduced 3T3-L1 adipocyte differentiation and lipid accumulation by suppressing GPDH activity. Furthermore, the anti-obesity effects can reduce body fat accumulation in adipose tissue, which caused a reduction in body weight gain. Therefore, the Mei-Gin formula is a promising candidate for the treatment of obesity; however, further investigation on the mechanistic pathway of lipolysis, oxidation, synthesis of fatty acid, and thermogenesis in adipose tissues in the future may provide deeper insight into the molecular mechanisms underlying obesity and help develop emerging therapeutic options.

## Figures and Tables

**Table 1:** Effects of Mei-Gin formulas on body weights, final body weight, and weight change in high-fat diet-induced obese rats.

Dietary Groups	Initial Body Weight (g)	Final Body Weight (g)	Weight Change (g)
ND	209 ± 2 ^a^	512 ± 17 ^c^	304 ± 17 ^c^
HFD	210 ± 2 ^a^	617 ± 16 ^a^	406 ± 16 ^a^
HFD + JMG-LD	210 ± 2 ^a^	563 ± 13 ^bc^	353 ± 13 ^bc^
HFD + JMG-HD	209 ± 2 ^a^	584 ± 13 ^ab^	375 ± 14 ^ab^
HFD + MGF-3-LD	209 ± 2 ^a^	599 ± 22 ^ab^	390 ± 20 ^ab^
HFD + MGF-3HD	209 ± 2 ^a^	559 ± 24 ^bc^	350 ± 23 ^bc^
HFD + MGF-7-LD	209 ± 2 ^a^	560 ± 18 ^bc^	351 ± 17 ^bc^
HFD + MGF-7-HD	209 ± 2 ^a^	548 ± 18 ^bc^	338 ± 18 ^bc^
HFD + PC	209 ± 2 ^a^	551 ± 15 ^bc^	341 ± 15 ^bc^

All values are presented as mean ± SEM. The values with different letters indicated a significant change between groups as determined by one-way ANOVA with post hoc Duncan’s test. Differences were considered statistically significant when *p* < 0.05. Weight change (g) = final body weight (g) − initial body weight (g).

**Table 2:** Effects of Mei-Gin formulas on feed intake, energy intake, feed efficiency, and water intake in high-fat diet-induced obese rats.

Dietary Groups	Feed Intake (g/Rat/Day)	Energy Intake (kcal/Rat/Day)	Feed Efficiency (%)	Water Intake (mL/Rat/Day)
ND	25 ± 1 ^a^	101 ± 2 ^b^	21 ± 1 ^c^	33 ± 1 ^bc^
HFD	21 ± 0 ^b^	110 ± 2 ^a^	35 ± 1 ^a^	36 ± 1 ^bc^
HFD + JMG-LD	19 ± 0 ^c^	101 ± 2 ^b^	33 ± 1 ^ab^	40 ± 1 ^a^
HFD + JMG-HD	20 ± 0 ^bc^	106 ± 2 ^ab^	33 ± 1 ^ab^	36 ± 2 ^bc^
HFD + MGF-3-LD	21 ± 1 ^b^	109 ± 3 ^a^	33 ± 2 ^ab^	34 ± 1 ^ab^
HFD + MGF-3HD	20 ± 1 ^bc^	106 ± 3 ^ab^	31 ± 2 ^b^	33 ± 1 ^c^
HFD + MGF-7-LD	20 ± 1 ^bc^	105 ± 2 ^ab^	31 ± 1 ^ab^	36 ± 1 ^bc^
HFD + MGF-7-HD	20 ± 0 ^bc^	104 ± 2 ^ab^	30 ± 2 ^b^	35 ± 1 ^bc^
HFD + PC	21 ± 0 ^bc^	107 ± 1 ^a^	30 ± 1 ^b^	35 ± 0 ^bc^

All values are presented as mean ± SEM. The values with different letters indicated a significant change between groups as determined by one-way ANOVA with post hoc Duncan’s test. Differences were considered statistically significant when *p* < 0.05. Feed efficiency (%) = [weight change (g)/total feed intake (g)] × 100%.

**Table 3:** Effects of Mei-Gin formulas on the weights of organs in high-fat diet-induced obese rats.

Dietary Groups	Organ Weights (g/Rat)				
	Heart	Liver	Spleen	Lung	Kidney
ND	2 ± 0 ^a^	15 ± 1 ^b^	1 ± 0 ^a^	2 ± 1 ^a^	3 ± 0 ^a^
HFD	2 ± 0 ^a^	19 ± 1 ^a^	1 ± 0 ^a^	2 ± 0 ^a^	4 ± 0 ^a^
HFD + JMG-LD	2 ± 0 ^a^	16 ± 1 ^b^	1 ± 0 ^a^	2 ± 0 ^a^	4 ± 0 ^a^
HFD + JMG-HD	2 ± 0 ^a^	17 ± 1 ^a b^	1 ± 0 ^a^	2 ± 0 ^a^	4 ± 0 ^a^
HFD + MGF-3-LD	2 ± 0 ^a^	17 ± 1 ^a b^	1 ± 0 ^a^	2 ± 0 ^a^	4 ± 0 ^a^
HFD + MGF-3-HD	2 ± 0 ^a^	16 ± 1 ^b^	1 ± 0 ^a^	2 ± 0 ^a^	4 ± 0 ^a^
HFD + MGF-7-LD	2 ± 0 ^a^	15 ± 1 ^b^	1 ± 0 ^a^	2 ± 0 ^a^	3 ± 0 ^a^
HFD + MGF-7-HD	2 ± 0 ^a^	15 ± 1 ^b^	1 ± 0 ^a^	2 ± 0 ^a^	3 ± 0 ^a^
HFD + PC	2 ± 0 ^a^	15 ± 1 ^b^	1 ± 0 ^a^	2 ± 0 ^a^	4 ± 0 ^a^

All values are presented as mean ± SEM. The values with different letters indicated a significant change between groups as determined by one-way ANOVA with post hoc Duncan’s test. Differences were considered statistically significant when *p* < 0.05.

**Table 4:** Effects of Mei-Gin formulas on the weights of perirenal adipose tissue, epididymal adipose tissue, mesenteric adipose tissue, retroperitoneal adipose tissue, and inguinal adipose in high-fat diet-induced obese rats.

Dietary Groups	Weights (mg/g Rat)				
	Perirenal Adipose Tissue	Epididymal Adipose Tissue	Mesenteric Adipose Tissue	Retroperitoneal Adipose Tissue	Inguinal Adipose Tissue
ND	28 ± 3 ^c^	23 ± 2 ^b^	20 ± 1 ^c^	21 ± 2 ^c^	14 ± 2 ^b^
HFD	48 ± 2 ^a^	32 ± 2 ^a^	31 ± 2 ^a^	36 ± 2 ^a^	21 ± 1 ^a^
HFD + JMG-LD	39 ± 2 ^b^	31 ± 2 ^a^	25 ± 1 ^b^	24 ± 2 ^bc^	16 ± 1 ^b^
HFD + JMG-HD	41 ± 2 ^ab^	32 ± 2 ^a^	27 ± 2 ^ab^	30 ± 4 ^abc^	17 ± 2 ^ab^
HFD + MGF-3-LD	43 ± 2 ^ab^	31 ± 2 ^a^	27 ± 2 ^ab^	32 ± 3 ^ab^	18 ± 2 ^ab^
HFD + MGF-3-HD	39 ± 3 ^b^	30 ± 2 ^a^	24 ± 3 ^bc^	25 ± 3 ^bc^	15 ± 2 ^b^
HFD + MGF-7-LD	39 ± 2 ^b^	30 ± 2 ^a^	25 ± 2 ^bc^	24 ± 3 ^bc^	13 ± 1 ^b^
HFD + MGF-7-HD	36 ± 2 ^b^	29 ± 2 ^a^	24 ± 2 ^bc^	24 ± 2 ^bc^	13 ± 1 ^b^
HFD + PC	38 ± 3 ^b^	30 ± 2 ^a^	25 ± 1 ^bc^	27 ± 3 ^bc^	14 ± 1 ^b^

All values are presented as mean ± SEM. The values with different letters indicated a significant change between groups as determined by one-way ANOVA with post hoc Duncan’s test. Differences were considered statistically significant when *p* < 0.05.

**Table 5:** Effects of Mei-Gin formulas on the weights of total body fat and adipose tissue in high-fat diet-induced obese rats.

Dietary Groups	Weights (mg/g Rat)		
	Visceral Adipose Tissue	Subcutaneous Adipose Tissue	Total Body Fat
ND	71 ± 5 ^c^	35 ± 3 ^d^	106 ± 7 ^c^
HFD	111 ± 5 ^a^	57 ± 3 ^a^	168 ± 7 ^a^
HFD + JMG-LD	96 ± 4 ^ab^	40 ± 2 ^bcd^	135 ± 5 ^b^
HFD + JMG-HD	100 ± 5 ^ab^	47 ± 5 ^abc^	146 ± 10 ^ab^
HFD + MGF-3-LD	100 ± 6 ^ab^	50 ± 4 ^ab^	150 ± 10 ^ab^
HFD + MGF-3-HD	93 ± 7 ^b^	40 ± 4 ^bcd^	132 ± 11 ^b^
HFD + MGF-7-LD	94 ± 5 ^b^	37 ± 3 ^cd^	131 ± 7 ^b^
HFD + MGF-7-HD	88 ± 6 ^b^	37 ± 3 ^cd^	126 ± 8 ^bc^
HFD + PC	92 ± 5 ^b^	41 ± 4 ^bcd^	134 ± 8 ^b^

All values are presented as mean ± SEM. The values with different letters indicated a significant change between groups as determined by one-way ANOVA with post hoc Duncan’s test. Differences were considered statistically significant when *p* < 0.05. Total body fat (mg/g rat) [visceral adipose tissue (mg) subcutaneous adipose tissue (mg)] ÷ final body weight (g). Visceral adipose tissue (mg/g rat) = [perirenal adipose tissue (mg) + epididymal adipose tissue (mg) + mesenteric adipose tissue (mg)] ÷ final body weight (g). Subcutaneous adipose tissue (mg/g rat) = [retroperitoneal adipose tissue (mg) + inguinal adipose tissue (mg)] + final body weight (g).

## Data Availability

Not applicable.
